# Moles of a Substance per Cell Is a Highly Informative Dosing Metric in Cell Culture

**DOI:** 10.1371/journal.pone.0132572

**Published:** 2015-07-14

**Authors:** Claire M. Doskey, Thomas J. van ‘t Erve, Brett A. Wagner, Garry R. Buettner

**Affiliations:** 1 Interdisciplinary Graduate Program in Human Toxicology, The University of Iowa, Iowa City, Iowa, 52242, United States of America; 2 Free Radical and Radiation Biology Program, Department of Radiation Oncology, The University of Iowa, Iowa City, Iowa, 52242, United States of America; Northwest Fisheries Science Center, NOAA Fisheries, UNITED STATES

## Abstract

**Background:**

The biological consequences upon exposure of cells in culture to a dose of xenobiotic are not only dependent on biological variables, but also the physical aspects of experiments e.g. cell number and media volume. Dependence on physical aspects is often overlooked due to the unrecognized ambiguity in the dominant metric used to express exposure, i.e. initial concentration of xenobiotic delivered to the culture medium over the cells. We hypothesize that for many xenobiotics, specifying dose as moles per cell will reduce this ambiguity. Dose as moles per cell can also provide additional information not easily obtainable with traditional dosing metrics.

**Methods:**

Here, 1,4-benzoquinone and oligomycin A are used as model compounds to investigate moles per cell as an informative dosing metric. Mechanistic insight into reactions with intracellular molecules, differences between sequential and bolus addition of xenobiotic and the influence of cell volume and protein content on toxicity are also investigated.

**Results:**

When the dose of 1,4-benzoquinone or oligomycin A was specified as moles per cell, toxicity was independent of the physical conditions used (number of cells, volume of medium). When using moles per cell as a dose-metric, direct quantitative comparisons can be made between biochemical or biological endpoints and the dose of xenobiotic applied. For example, the toxicity of 1,4-benzoquinone correlated inversely with intracellular volume for all five cell lines exposed (C6, MDA-MB231, A549, MIA PaCa-2, and HepG2).

**Conclusions:**

Moles per cell is a useful and informative dosing metric in cell culture. This dosing metric is a scalable parameter that: can reduce ambiguity between experiments having different physical conditions; provides additional mechanistic information; allows direct comparison between different cells; affords a more uniform platform for experimental design; addresses the important issue of repeatability of experimental results, and could increase the translatability of information gained from *in vitro* experiments.

## Introduction

In the testing of xenobiotics, medicines, and natural products for biochemical and biological responses, the use of laboratory animals is regarded as the best model for providing information to predict effects in humans. The U.S. National Institutes of Health (NIH), as well as other research institutions worldwide, are seeking to minimize the use of animals in this 21^st^ century by encouraging the development, validation, and implementation of non-animal based studies (NIH Revitalization Act of 1993 SEC.404C http://grants.nih.gov/grants/olaw/pl103-43.pdf as accessed 2015.03.31). To succeed, it is important to gain the maximum information possible from *in vitro* experiments with the goal to accurately predict biological effects in humans.

A critical element in the foundation of scientific research is reproducibility. This problem encompasses a wide array of issues ranging from statistical considerations, to laboratory standards, practices, and reporting (Principles and Guidelines for Reporting Preclinical Research at http://www.nih.gov/about/reporting-preclinical-research.htm as accessed 2015.03.31) [1] and references therein. Here we examine the topic of how to specify dose or exposure to a xenobiotic in cell culture experiments with the goal to address an aspect of the problem of reproducibility in science. This matter may also result in more successful translation of information from cell culture studies to whole organisms, thereby addressing the 3R’s, replacement, reduction and refinement, for the use of animals in research [2].

When assessing the biological consequences of xenobiotics in *in vitro* experiments, dose is a central parameter [3, 4]. Groothuis et al. have reviewed some of the major issues with dose and reproducibility of cell culture experiments and the translation of *in vitro* observations to *in vivo* models [5]. This instructive review examines various dose-metrics, including nominal concentration, total concentration, freely available concentration, as well as various dose-metrics for xenobiotics associated with cells. The most common dosing metric in cell culture experiments is the initial concentration, i.e. nominal concentration (e.g. mol L^-1^, g L^-1^; see [5].), of a compound added to the culture medium [6, 7, 8]. Using the nominal concentration of a xenobiotic as a measure of exposure can be unexpectedly problematic by yielding ambiguous information on the true exposure of cells to xenobiotics in cell culture experiments and provide limited mechanistic insights [9, 10, 11]. Exposure is highly dependent on the actual experimental conditions, e.g. volume of the medium used and total moles or mass of xenobiotic. This can lead to large variations in experimental results from unrecognized differences in the actual exposure due to changes in the physical conditions (e.g. volume of medium and number of cells used) of experiments. This is especially important with the introduction of high-throughput screening techniques [12, 13]. In these techniques, low volumes of media coupled with low cell numbers in multi-well plates result in many changes in physical parameters compared to traditional cell culture vessels, e.g. cell culture dishes and flasks.

The objective of this research is to re-evaluate how dose is considered and reported with the ultimate goal to reduce some of the ambiguity introduced by common dosing metrics. Here we examine the value of a complementary but different dosing-metric, i.e. moles of xenobiotic applied normalized to the number of cells present at the time of exposure (i.e. counted cells). We **hypothesize** that for many xenobiotics, using **moles per cell** (mol cell^-1^) as a dosing metric will reduce the dependency of experimental results on physical parameters. Although this dosing metric does not directly indicate how much compound is in, or associated with, the cell, it provides the advantage of being readily implementable with every assay by counting the number of cells prior to exposure in a representative cell culture vessel (e.g. cell culture well, dish, or flask) and taking into account the volume of medium over the cells.

Dose as mol cell^-1^ can also provide additional information not easily obtained by other dosing metrics. Many xenobiotics bring about irreversible changes in critical covalent bonds (targets) in cells; some form very stable complexes with cellular targets. Typically, a certain fraction of these targets must be transformed to bring about biochemical and biological consequences. For these agents, the actual information needed to unravel detailed mechanisms is: how many targets are there in a cell and how many molecules (moles) of xenobiotic per cell are required to transform a critical number of targets that will result in a biochemical and biological response? We **hypothesize** that using **mol cell** ^**-1**^ as the dosing metric in cell culture experiments will give researchers this type of information, thereby expanding the information that can be extracted from the data obtained from cell culture experiments.

To assess these hypotheses, the effects of the electrophile 1,4-benzoquinone (1,4-BQ) and oligomycin A, which forms a highly stable complex with mitochondrial complex V, were investigated in a variety of cell lines. Using these compounds, mol cell^-1^ was investigated as a metric for: quantitative causality; the consequences of sequential vs. bolus additions; and the influence of intracellular volume on biological responses.

We anticipate that by specifying dose as mol cell^-1^, reproducibility of results from *in vitro* experiments across different laboratories will be improved; additional information will be available from cell culture data; and translation of this information to whole organisms will be more successful.

## Materials and Methods

### Materials

1,4-Benzoquinone, dimethyl sulfoxide, oligomycin A, adenosine 5’-triphosphate (ATP) disodium salt hydrate, 1-octane-sulfonic acid, sodium phosphate, acetonitrile, glutathione, glutathione disulfide, and diethylenetriaminepentaacetic acid were obtained from Sigma Aldrich (St. Louis, MO, USA).

### Cell lines

MIA PaCa-2, C6, HepG2, MDA-MB231, and A549 cells were purchased from American Type Culture Collection (Manassas, VA). Origin and bio-physical parameters of each cell are listed in Table 1. All cells were cultured in Dulbecco’s modified eagle medium (DMEM) with high glucose from Invitrogen (Grand Island, NY), supplemented with 10% fetal bovine serum (FBS) and Penicillin Streptomycin (100 units mL^-1^) at 37°C, 5% CO_2_. Sufficient medium was prepared to complete an experiment, including all replicates. All media preparations for a set of experiments contained FBS from the same lot number to minimize variation between experiments.

**Table 1:** Physical and biological parameters of cell lines used.

Cell Line	Type^a^	Doubling time	Literature intracellular Volume^b^	Measured Intracellular Volume	Protein mass per cell^c^
		(h)	(pL)	(pL)	(pg)
C6	Rat glioma	22 [14]	1.08 [15]	1.04 (0.12)^d^ ^,^ ^e^	141 (26)^e^
	CCL-107			0.95 (0.07)^f^ ^,^ ^e^	
MDA-MB231	Human mammary adenocarcinoma	34^g^	1.53 [16]	2.37 (0.01)^d^ ^,^ ^e^	415 (16)
	HTB-26			2.21 (0.14)^f^ ^,^ ^e^	
A549	Adenocarcinoma alveolar epithelial	22^h^	1.76 [15]	2.38 (0.01)^d^ ^,^ ^e^	659 (15)
	CCL-185			2.69 (0.03)^f^ ^,^ ^e^	
MIA PaCa-2	Human pancreatic carcinoma	24^g^	2.03 [16]	2.61 (0.02)^d^ ^,^ ^e^	757 (59)
	CRL-1420			2.10 (0.02)^f^ ^,^ ^e^	
HepG2	Human hepatoma cells	42^g^	2.54 [17]	2.83 (0.09)^d^ ^,^ ^e^	1192 (186)
	HB-8065			2.96 (0.07)^f^ ^,^ ^e^	

^a^ Type and ATCC #

^b^ Literature values for intracellular volume of cell lines.

^c^ Protein mass per cell was measured in trypsinized cells using SDS-Lowry protein assay, *n* = 3 for each cell line. Albumin from bovine serum (Sigma Aldrich; Cohn Fraction V, Sigma-A2153) was used as a standard [18].

^d^ As measured with Moxi Z Mini Automated Cell Counter (ORFLO Technologies), *n* = 3 for each cell line. Presented is the mean of triplicate biological samples.

^e^ Standard error of the mean of three triplicate biological samples.

^f^ As measured with Z2 Coulter Counter, *n* = 3 for each cell line. Presented is the mean of triplicate biological samples.

^g^ As measured in triplicate under cell culture conditions reported here.

^h^ ATCC http://www.atcc.org/CulturesandProducts/CellBiology/CellLinesandHybridomas/tabid/169/Default.aspx as accessed on 2015-05-29.

The volumes of MIA PaCa-2, C6, HepG2, MDA-MB231, and A549 cells were determined in triplicate using both a Moxi Z Mini Automated Cell Counter (ORFLO) and a Z2 Coulter Counter (Beckman Coulter, Inc.). Cells were trypsinized, collected in PBS, and centrifuged to pellet. Cell pellets were then suspended in ISOTON II Diluent (Beckman Coulter, Inc.) and the cell size (intracellular volume) was immediately determined using both cell sizing methods.

Protein mass per cell was measured in each cell line (i.e. MIA PaCa-2, C6, HepG2, MDA-MB231, and A549 cells) using the SDS-Lowry protein assay, *n* = 3 for each cell line. Cells were plated in triplicate 60 mm x 15 mm culture dishes at equal densities (500,000 cells per dish). Cells were allowed to adhere and grow 48 h after plating. Cells were then trypsinized, collected in PBS, centrifuged (193 ***g***) and suspended in a small volume of PBS (200 μM). A portion of the cell pellet was used for counting with the hemocytometer, so the total number of the cells in each pellet was known. Cell pellets were kept in -80°C until time of analysis. Prior to protein mass determination, cell pellets were thawed and sonicated for 1 min to lyse cells. Protein mass per cell was measured using the SDS-Lowry and cell counts allowed for the determination of protein mass per cell in each sample. Albumin from bovine serum (Sigma Aldrich; Cohn Fraction V, Sigma-A2153) was used as a standard [18].

### Exposure to 1,4-benzoquinone

MIA PaCa-2, C6, HepG2, MDA-MB231, and A549 cells were seeded into multiple 25 cm^2^ or 75 cm^2^ culture flasks at equal density and allowed to grow until ≈70% confluent. One of the flasks was used strictly for calculating the initial dose in units of mol cell^-1^. To achieve this, prior to exposure to 1,4-BQ, cells were counted in this flask with a hemocytometer; this number of total cells, which were present immediately prior to exposure, was used to calculate the initial (applied) dose in units of mol cell^-1^. Exposure media were prepared by addition of 1,4-BQ stock solution in DMSO to fresh culture media and vigorous mixing. All cell lines were cultured in and exposed in DMEM high glucose with 10% FBS and 0.8% penicillin streptomycin. Growth medium was exchanged with the exposure medium containing 1,4-BQ/DMSO or DMSO alone (vehicle control). Exposures to 1,4-BQ ranged from 0 to 2000 femtomol cell^-1^ (femto = 10^−15^; abbreviation = fmol cell^-1^), i.e. 0 to 320 μM under the physical conditions of these experiments. Cells were then incubated for 4 h at 37°C, 5% CO_2_. For control experiments, 2% DMSO (0.28 M) was added to fresh culture medium so that the percentage DMSO was equivalent to that of cells exposed to 1,4-BQ. For a single experimental protocol where media volumes were varied within the experiment, the percentage of DMSO in fresh culture medium varied from 0.25–4% (0.035 to 0.51 M); control experiments were done using the highest and lowest concentrations of DMSO and no toxicity was observed.

### Exposure to oligomycin A

MIA PaCa-2 cells were seeded at varying cell densities (25,000 cells–400,000 cells) in duplicate 6-well culture plates and allowed to adhere and grow for 48 h before exposure to oligomycin A. Prior to exposure to oligomycin A, the number of cells per well of the duplicate 6-well plate was determined with a hemocytometer to ascertain the number of cells in each well for the varying cell densities; this number of total cells, present immediately prior to exposure, was used to calculate the initial dose in units of mol cell^-1^. Exposure media were prepared by addition of oligomycin A stock solution in DMSO to fresh culture media. Growth medium was exchanged with the exposure medium containing oligomycin A (2 μM) in DMSO or DMSO alone (vehicle control). All controls and treatments had 0.3% DMSO (0.039 M) in the medium. Exposures to oligomycin A ranged from 0 to 87 fmol cell^-1^ (0 μM in control treatments and 2 μM for oligomycin treatments), depending on the cell density. Cells were exposed to oligomycin A for 1 h prior to measurement of intracellular ATP.

### Intracellular ATP assay

A cell suspension (100 μL, 50,000 cells) was added to each well in an opaque-walled, 96-well plate. To this, 100 μL of reagent from an ATP kit (Promega, CellTiterGlo) was added to lyse the cells and initiate the luminescence reaction. After 10 min, luminescence was measured on a microplate reader. ATP standard curves with concentrations between 0–1000 μM were generated for each experiment. The ATP concentration was determined from the corresponding standard curve and converted to an intracellular concentration using the cell number and cell volume, as done for intracellular GSH concentrations [19, 20].

### Clonogenic survival assay

To assess the cytotoxicity of exposure to 1,4-BQ, cells were plated for a clonogenic assay following the 4-h exposure to 1,4-BQ. The exposure medium was removed, cells trypsinized and counted with a hemocytometer and plated at a cell density of 200 and 400 cells in 3.0 mL of medium in 60 mm^2^ dishes, with the exception of HepG2 cells, which were plated at 2000 and 5000 cells per dish. Plates were incubated for 6–14 days at 37°C, 5% CO_2_. After the growth period, cells were fixed with 70% ethanol and stained with Coomassie Blue. Colonies were counted as a grouping of 50 or more cells. The plating efficiency and surviving fraction were determined [21]; plating efficiency (PE) = (colonies counted / cells plated) x 100; survival fraction = (PE of treated sample / PE of control) x 100. From plots of clonogenic survival fraction vs. dose of 1,4-BQ, the Effective Dose 50 (ED_50_, 50% clonogenic survival) was determined.

### Trypan blue staining

As a measure of cell viability the trypan blue exclusion assay was employed [22]. Trypan blue (10 μL of 0.4%) was added to 10 μL of a cell suspension from each exposure. Using 10 μL of this cell suspension, a hemocytometer was used to count stained and unstained cells; cell viability = (unstained cells / total cells) x 100.

### Exposure of cells for GSH and GSSG determination

To assess the effect of exposure to 1,4-BQ on intracellular levels of GSH and GSSG, MIA PaCa-2 cells were cultured to ≈70% confluence in 125 cm^2^ flasks. Prior to exposure, the growth media were exchanged with exposure media as described above. Cells were exposed to 1,4-BQ at increasing mol cell^-1^ (0 to 500 fmol cell^-1^) for a period of 30 min at 37°C, 5% CO_2_. Also, additional experiments with a bolus mol cell^-1^ of 1,4-BQ (6.1 fmol cell^-1^) for up to 24 h were performed with cells harvested at 0, 10, 60, 400, and 1400 min after exposure. All cells in a flask, with the exception of a minute quantity for cell counting, were used to allow for the measurement of GSSG.

### GSH and GSSG determination with HPLC-BDD

Cells were removed from culture dishes with trypsin/EDTA. Samples were centrifuged and supernatant removed. Cell pellets were resuspended in 5% perchloric acid (PCA) containing 100 μM diethylenetriaminepentaacetic acid (DTPA, alias DETAPAC). The processed samples were stored at -80°C until the day of analysis. Prior to analysis samples were thawed and centrifuged to remove the precipitated proteins.

To determine the amount of GSH and GSSG in cultured cells, HPLC with electrochemical detection (ESA CoulArray with a temperature-controlled (4°C) auto-sampler) was used following the protocol outlined by Park et al. [23]. The method is based on an electrochemical detection (ECD) system using a boron-doped diamond disc (BDD) electrode (Model 5040, ESA Biosciences, Chelmsford, MA, USA). Samples were loaded into auto-sampler vials with a 100 μL glass insert. For analysis a sample was loaded on the column and eluted isocratically with 97% 25 mM sodium phosphate, pH 2.65, 1.4 mM 1-octane-sulfonic acid / 3% acetonitrile for 60 min. With each set of samples, four standards containing GSH (500–8000 pmol on column) and GSSG (200–2000 pmol on column) were included; these standard curves were used to quantify analytes for that particular sample set. Quantitation was performed by integrating the GSH and GSSG peaks in the BDD electrode channel with ESA Coularray for Windows version 1.12.

The GSH amount (mol) was divided by the number of cells associated with the sample (counted prior to resuspension in perchloric acid/DTPA using a hemocytometer) giving a value of mol cell^-1^. The mol cell^-1^ value was then divided by the intracellular volume resulting in an average intracellular concentration of mol L^-1^ for GSH and GSSG [20].

### Statistics

Results are expressed as the mean ± SEM. Statistical analyses were performed using One-way analysis of variance and where appropriate the unpaired student’s t test; *p* values less than 0.05 were considered statistically significant. Calculations were performed using IBM SPSS Statistics for Windows, Version 20.0 (IBM Corp., Armonk, NY).

## Results

To test our hypotheses that expressing dose as mol cell^-1^ will: yield more information; lead to improved experimental design; and better predict biochemical and biological responses, 1,4-benzoquinone (1,4-BQ) and oligomycin A were used as model xenobiotics. 1,4-Benzoquinone is an electrophilic quinone that readily forms covalent bonds with amine- and thiol-containing biomolecules (S1 Fig) while oligomycin A inhibits mitochondrial ATP production by forming a quite stable complex with mitochondrial complex V [24]. Because these two xenobiotics have quite different chemistry they can serve as representative candidates to address our hypotheses.

### Comparison of dosing metrics, 1,4-benzoquinone as an example

**Fig 1:**
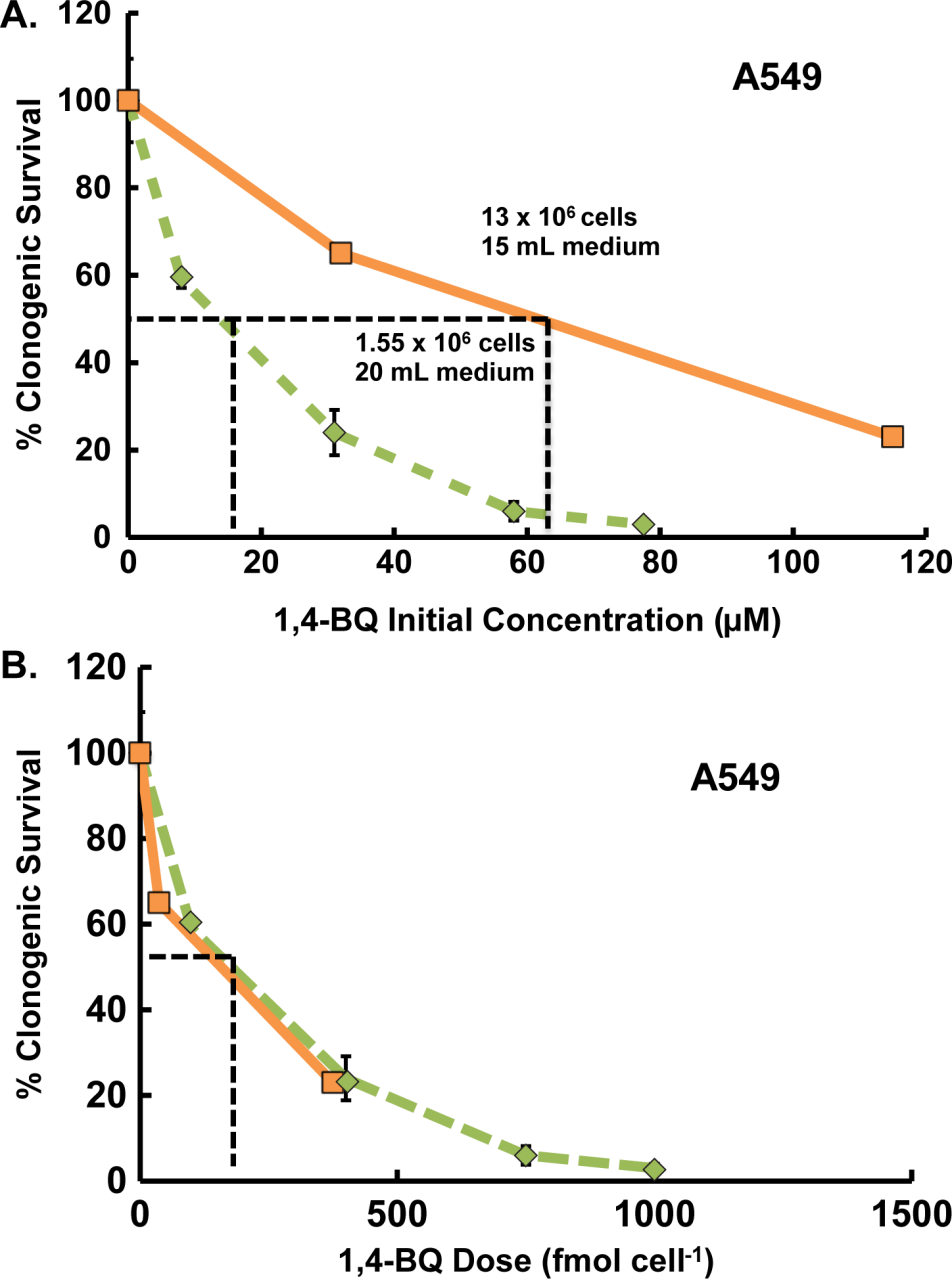
Dose of 1,4-BQ expressed as mol cell^-1^ allows direct comparisons between different experimental conditions and more accurately reports toxicity than initial concentration in medium. Clonogenic survival of A549 cells was observed after a 4-h exposure to 1,4-BQ using two different experimental conditions: orange square, 13 x 10^6^ cells exposed in 15.0 mL of medium; green diamond, 1.55 x 10^6^ cells exposed in 20.0 mL medium. The doses are expressed in: **(A)** Initial concentration in medium (μM), note that EC_50_ depends on the physical setup of the experiment; **(B)** Mol cell^-1^ basis (here fmol cell^-1^); note that ED_50_ is independent of the physical setup of the experiment. The two clonogenic survival curves are representative experiments with each point being the median of six plates. Error bars represent the standard error of the median; many error bars are smaller than the symbol.

To evaluate the differences between expressing exposure as mol cell^-1^ compared to the initial concentration, A549 cells were exposed to 1,4-BQ for 4 h under two physically different experimental conditions, Fig 1. Toxicity was assessed using clonogenic assays (reproductive cell death). The physical conditions that were varied between the two experimental set-ups were the volume of medium and number of cells. Between the two experimental set-ups there was an ≈8-fold difference in the number of cells used, but only a 0.75-fold difference in the volume of medium used, yielding two very different exposures to 1,4-BQ. When exposure is expressed as the initial, i.e. nominal concentration of 1,4-BQ in the medium, the percent survival *vs*. dose for the two conditions were statistically very different; EC_50_ (Effective Concentration yielding 50% survival) values are 14 and 62 μM, Fig 1A. However, when dose is expressed as fmol cell^-1^, the values of ED_50_ (Effective Dose 50% survival) for the two experimental conditions are essentially the same, 160 and 170 fmol cell^-1^, Fig 1B.

**Fig 2:**
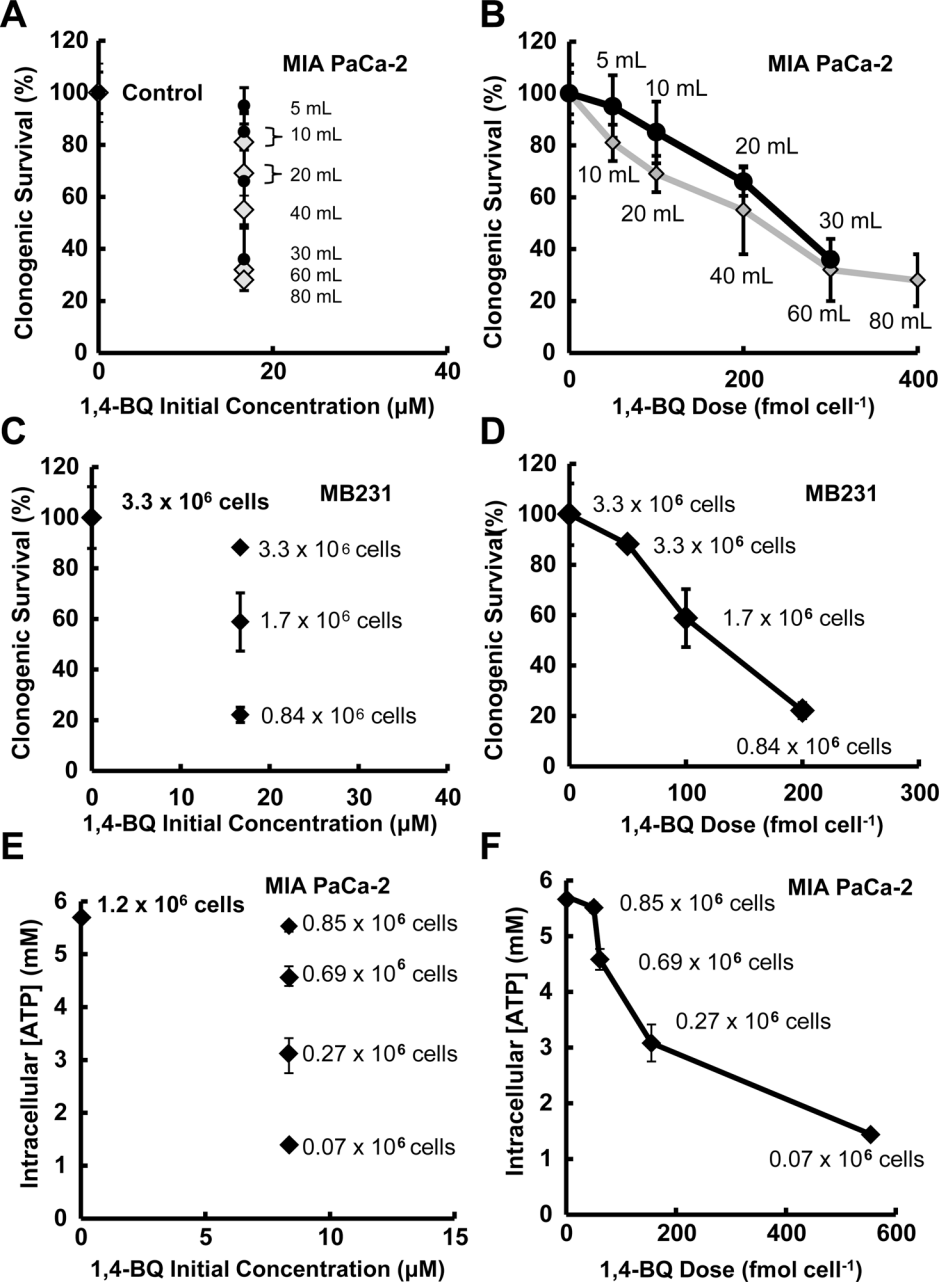
Dose specified as mol cell^-1^ more accurately reports toxicity of 1,4-BQ in cell culture experiments than initial concentration in medium. **(A)** These data show two different physical setups for the experiments, black circle (1.7 x 10^6^ cells) and gray diamond (3.3 x 10^6^ cells). Clonogenic survival of MIA PaCa-2 cells was measured in triplicate after a 4-h exposure to 16.7 μM 1,4-BQ in different volumes of medium, yielding a range of doses on a mol per cell basis. No dose dependence is observed when the dose 1,4-BQ is expressed as the initial concentration in the culture medium. Error bars represent the standard deviation of the median of triplicate measures. **(B)** The data of panel **A** are transformed to express the dose of 1,4-BQ in units of fmol cell^-1^. A far better delineation of the dose-dependent toxicity is observed in each of the two sets of experiments. Error bars represent the standard deviation of the median of triplicate measures. **(C)** Using a varying number of MDA-MB231 cells, clonogenic survival varied considerably after a 4-h exposure to 16.7 μM 1,4-BQ in 10.0 mL of medium in T-25 culture flasks (each point has *n* = 2 for biological replicates, *n* = 3 within each replicate). Error bars represent the standard deviation of the mean. **(D)** As with MIA PaCa-2 cells, when the dose of 1,4-BQ is expressed in units of fmol cell^-1^, a much more informative delineation of the dose-dependent toxicity in MDA-MB231 cells is observed. Error bars represent the standard deviation of the mean. (**E**) Using a varying number of MIA PaCa-2 cells, intracellular ATP concentration of MIA PaCa-2 cells varied considerably after a 4-h exposure to 8.35 μM 1,4-BQ in 5.0 mL of medium in T-25 culture flasks (each point has a *n* = 2 for biological replicates, *n* = 2 within each replicate). Error bars represent the standard deviation of the mean; many standard deviations are smaller than the symbol. **(F)** When the dose of 1,4-BQ is expressed in units of fmol cell^-1^, a much more informative delineation of the dose-dependent effect on intracellular ATP concentration in MIA PaCa-2 cells is observed. Error bars represent the standard deviation of the mean.

The ambiguity present in expressing dose in terms of initial concentration compared to mol cell^-1^ was further explored in the experiments of Fig 2 panels A-D. In the experiments of Fig 2, panels A and B, the initial concentration of 1,4-BQ was constant for all exposures (16.7 μM); however, mol cell^-1^ was varied for each experiment by exposing an identical number of MIA PaCa-2 cells in varying volumes of medium (5.0 mL to 30.0 mL or 10.0 mL to 80.0 mL). Clonogenic survival varied substantially as the volume of medium varied, even though the initial concentration in medium was identical throughout all exposures, Fig 2A. In contrast, there is clear dose-dependence when dose is specified in mol cell^-1^, Fig 2B. In this experiment, mol cell^-1^ provides an unambiguous dosing metric that more accurately reflects the exposure as opposed to expressing dose as the initial added concentration of 1,4-BQ.

Next, the number of MB231 cells exposed was varied, but in all treatments identical volumes of medium (10.0 mL medium) and identical concentrations of 1,4-BQ (16.7 μM) were used. Again, clonogenic survival was different in each experiment, even though the dose in terms of initial concentration in the medium was identical in all exposures, Fig 2C. In contrast, expressing dose in mol cell^-1^ rather than the initial concentration throughout these experiments better reflects the cellular exposure and clearly shows an anticipated dose-dependence, Fig 2D.

ATP content per cell was used as an alternative endpoint after exposure of MIA PaCa-2 cells to 1,4-BQ, Fig 2 panels E and F. As with the biological endpoint of clonogenic survival, ATP content varied substantially as the number of cells exposed varies, even though the initial concentration of 1,4-BQ in medium was identical throughout all exposures, Fig 2E. However, there is the anticipated dose-dependence when dose is specified in mol cell^-1^, Fig 2F. In these experiments, mol cell^-1^ of 1,4-BQ provides an unambiguous dosing metric that better reflects the exposure to a xenobiotic and relates to both biochemical and biological consequences as opposed to expressing dose as the initial added concentration of 1,4-BQ.

**Fig 3:**
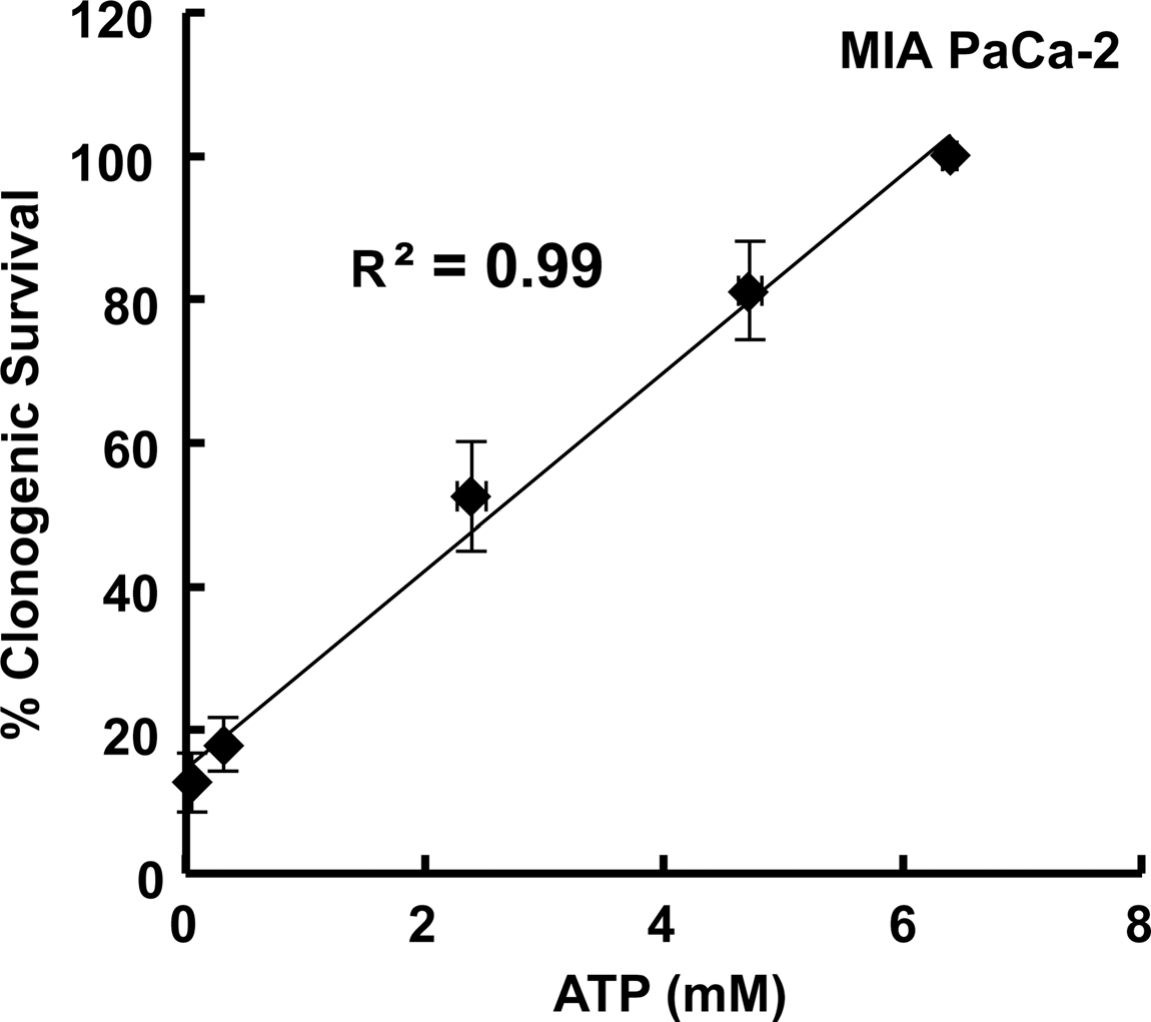
Clonogenic survival correlates directly with intracellular ATP concentration following exposure to 1,4-BQ. Clonogenic survival of MIA PaCa-2 cells following 4-h exposure to (0–1000 fmol cell^-1^) 1,4-BQ was plotted against intracellular ATP concentration of MIA PaCa-2 cells also following 4-h exposure to (0–1000 fmol cell^-1^) 1,4-BQ. Clonogenic survival directly correlates with intracellular ATP concentration following 4-h exposure to 1,4-BQ. Clonogenic survival is presented as the mean of *n* = 3 biological replicates with error bars representing the standard error of the mean. Intracellular ATP is presented as the mean of *n* = 2 biological replicates with error bars representing the standard error of the mean. Some error bars are smaller than the symbols.

It has been demonstrated that decreased cellular ATP can correlate with decreases in cell viability and increases in pro-apoptotic markers, such as caspases [25]. In Fig 2, we see that changes in ATP content per cell followed closely with results of the clonogenic assays, i.e. lower cellular ATP after exposure to 1,4-BQ correlates with poorer survival. A plot of clonogenic survival *vs*. intracellular ATP concentration produces a remarkable linear relationship between a biochemical parameter and a biological consequence, Fig 3. This is as might be expected because each measure is a function of the exposure to 1,4-BQ as expressed in units of mol cell^-1^. That each measure is a quantitative function of applied dose as mol cell^-1^ opens a new window to examine mechanisms of toxicity.

### Comparison of dosing metrics, oligomycin A

**Fig 4:**
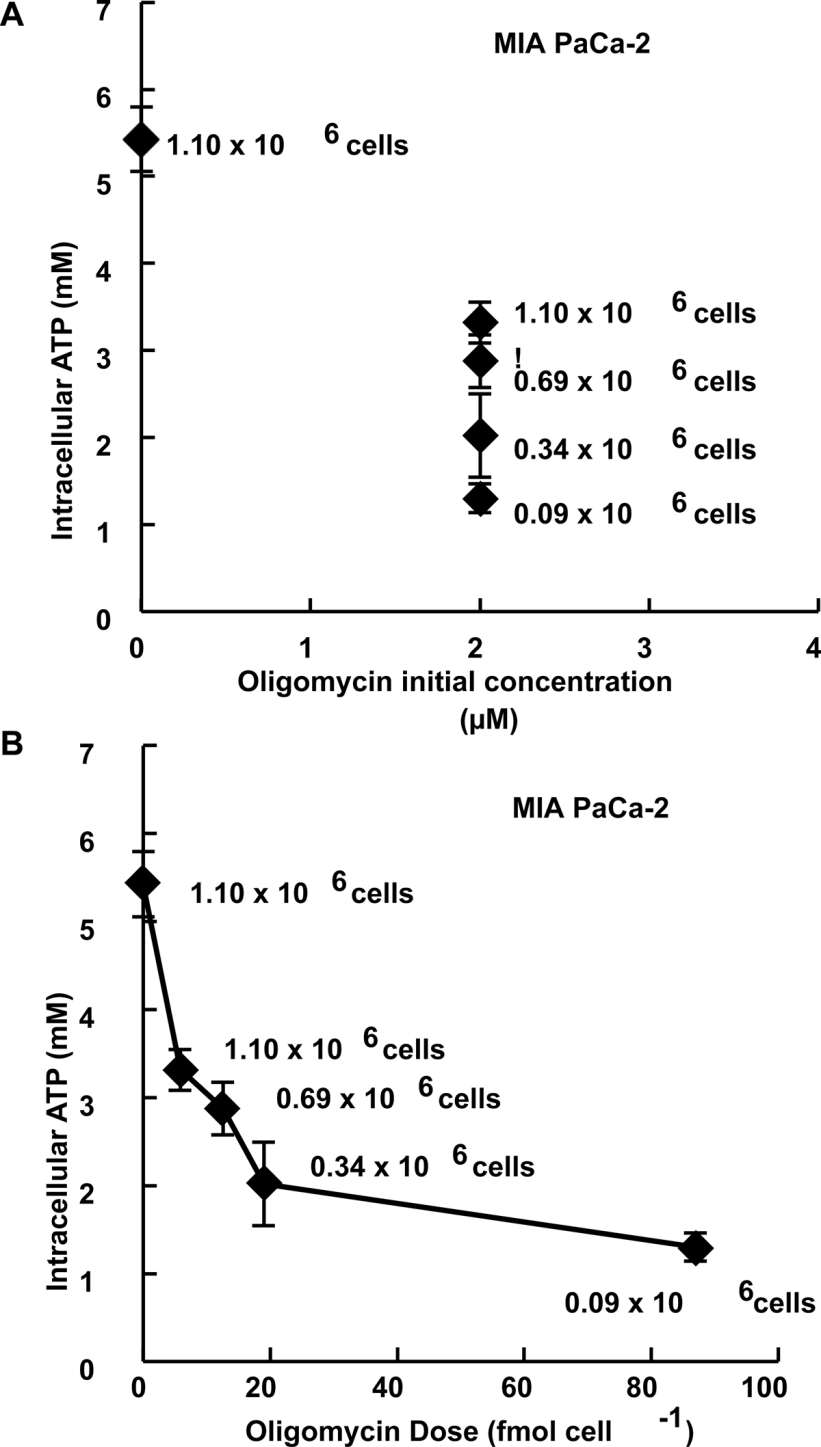
Expressing dose as mol cell^-1^ yields more information and can be helpful when using biochemical tools in cell culture experiments: ATP per cell decreases with increasing dose of oligomycin A on a per cell basis. The levels of ATP in MIA PaCa-2 cells were measured immediately after a 1-h exposure to oligomycin A. **(A)** ATP levels measured following a 1-h exposure of MIA PaCa-2 cells at varying cell densities to 2 μM oligomycin A in 3.0 mL medium. Doses of oligomycin A are expressed in initial concentration of oligomycin A in the medium (μM) (*n* = 4, error bars are standard deviation of the mean). **(B)** Doses of oligomycin A are expressed in mol cell^-1^ (fmol cell^-1^) (*n* = 4, error bars are standard deviation of the mean).

Oligomycin A is an antibiotic isolated from *Streptomyces diastatochromogenes*; it is an inhibitor of mitochondrial H^+^- ATP synthase, complex V of the electron transfer system. Oligomycin A is widely used as a biochemical tool for studying mitochondrial respiration and oxidative phosphorylation. It inhibits the H^+^-ATP synthase by binding to the oligomycin sensitivity-conferring protein (OSCP); at high concentrations oligomycin can also inhibit the Na^+^+K^+^-ATPase [24]. Unlike 1,4-BQ, oligomycin A does not covalently bind to its target, rather it forms a tight complex with OSCP and/or the Na^+^+K^+^-ATPase. It is a non-competitive inhibitor in its interaction with OSCP with a *V*_*max*_ = 0.77 μmol min^-1^ mg^-1^, *K*_*m*_ = 120 μM and *K*_*i*_ = 11 nM [26]. Oligomycin A has a dissociation constant *K*_*d*_ ≈ 10^−6^ M with the Na^+^-K^+^ ATPase [24, 26, 27]. Because oligomycin A forms a tight complex, we hypothesized that mol cell^-1^ would be a better metric for expressing dose than initial concentration. In Fig 4, panels A and B, the number of MIA PaCa-2 cells exposed to oligomycin A was varied (0.09 x 10^6^ to 1.1 x 10^6^ cells), but in all treatments cells were exposed to identical concentrations of oligomycin A (2 μM in 3.0 mL of medium). This achieves different amounts (moles) of oligomycin A on a per cell basis (0–87 fmol cell^-1^). ATP content was measured following exposure; the resulting steady-state intracellular ATP concentration was different after each treatment (*i*.*e*. different numbers of cells were exposed), even though the dose in terms of initial concentration in the medium was identical for all exposures, Fig 4 panels A and B. The expected dose-response was achieved when dose is expressed in terms of mol cell^-1^. These data demonstrate that the effects of xenobiotics that are popular biochemical tools could provide different results under various experimental conditions. This potential ambiguity can be overcome by using mol cell^-1^ as a standardized and reliable dosing metric when specifying how these tools were used.

### Sequential addition vs. bolus addition

**Fig 5:**
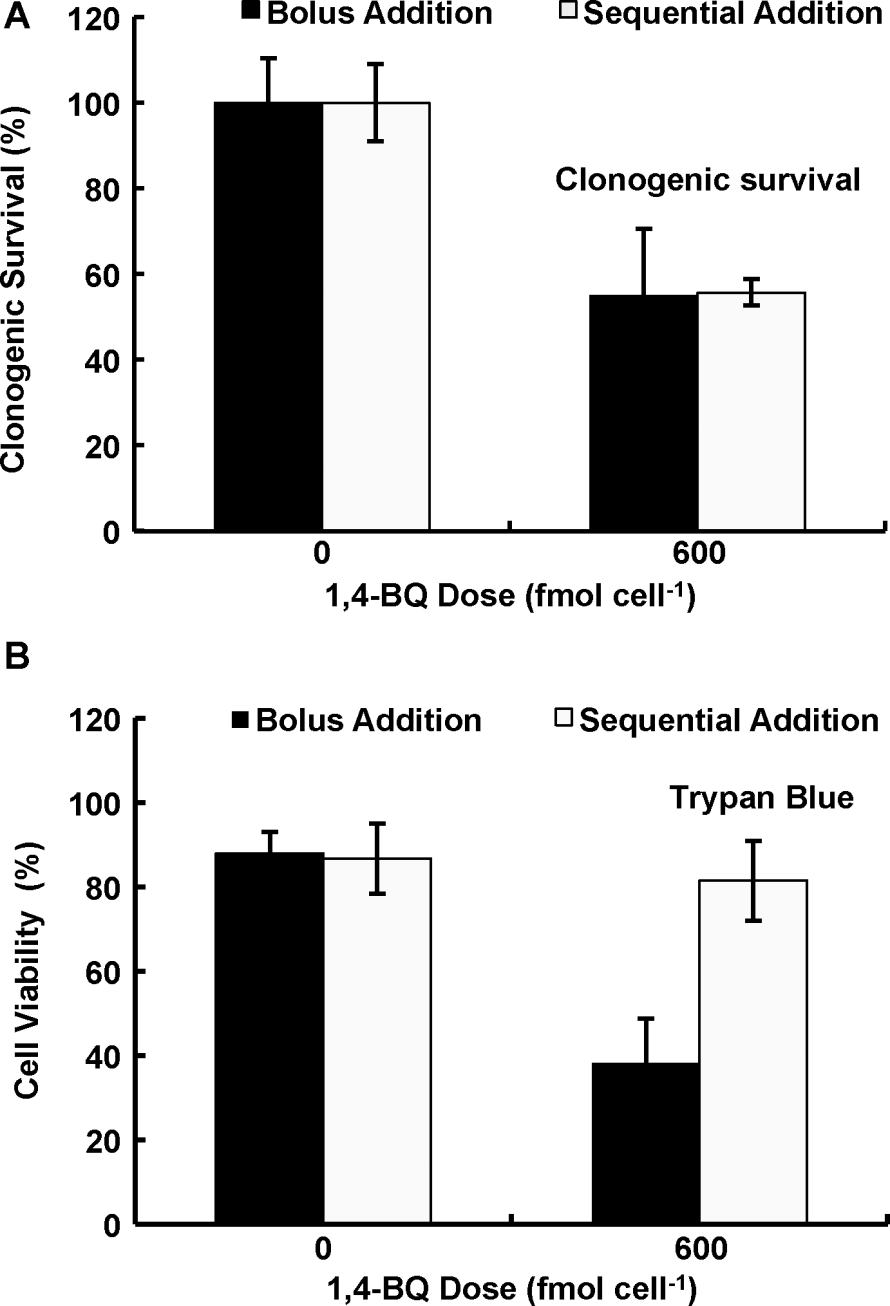
A single bolus addition or sequential additions of 1,4-BQ can provide differential toxicities based on the endpoint measured. **(A)** Clonogenic survival of MIA PaCa-2 cells was evaluated after a bolus addition of 600 fmol cell^-1^ of 1,4-BQ or incremental additions of 1,4-BQ every 20 min over the 4-h exposure period (12 separate but equal additions) to yield a total dose of 600 fmol cell^-1^. Controls represent additions of DMSO only to the culture media using protocols parallel to additions of 1,4-BQ. Clonogenic survival was the same for both exposure methods (*n* = 3, error bars are standard deviation of the mean). There is no statistical difference in the clonogenic survival between the two protocols. Each is different from the controls (*p* < 0.05). **(B)** Cell viability as indicated with trypan blue staining produced quite different results using a bolus dose of 1,4-BQ *vs*. sequential addition (*n* = 3, error bars are standard deviation of the mean). A single bolus addition produces a significant difference between control and sequential addition (*p* < 0.05). Whereas the sequential addition is the same as the control (*p* > 0.05).

Another challenge in dosing is the comparison between bolus *vs*. sequential addition of the xenobiotic. To examine the usefulness of mol cell^-1^ in this setting, several smaller doses of 1,4-BQ were added sequentially throughout a 4-h exposure time and compared to a single bolus addition producing the same total amount of 1,4-BQ. MIA PaCa-2 cells were exposed to sequential additions or a single bolus addition of 1,4-BQ for a total cumulative dose of 600 fmol cell^-1^, then clonogenic survival and trypan blue staining assays were performed, Fig 5. Identical toxicity was observed between a single bolus dose of 1,4-BQ (600 fmol cell^-1^) *versus* 12 sequential doses (50 fmol cell^-1^ per addition), Fig 5A. However, the trypan blue assay results showed statistically significant differences in cytotoxicity between the two dosing methods, Fig 5B. Thus, reproductive cell death is affected by the total dose of 1,4-BQ, whereas membrane integrity has an element of time and concentration because several smaller exposures over time appear to be less damaging than a single bolus dose; ultimate lethality was the same as seen by clonogenic survival pointing to the importance of the need to use an appropriate assay to provide the best information.

### Causality, census of agent and reaction targets

**Fig 6:**
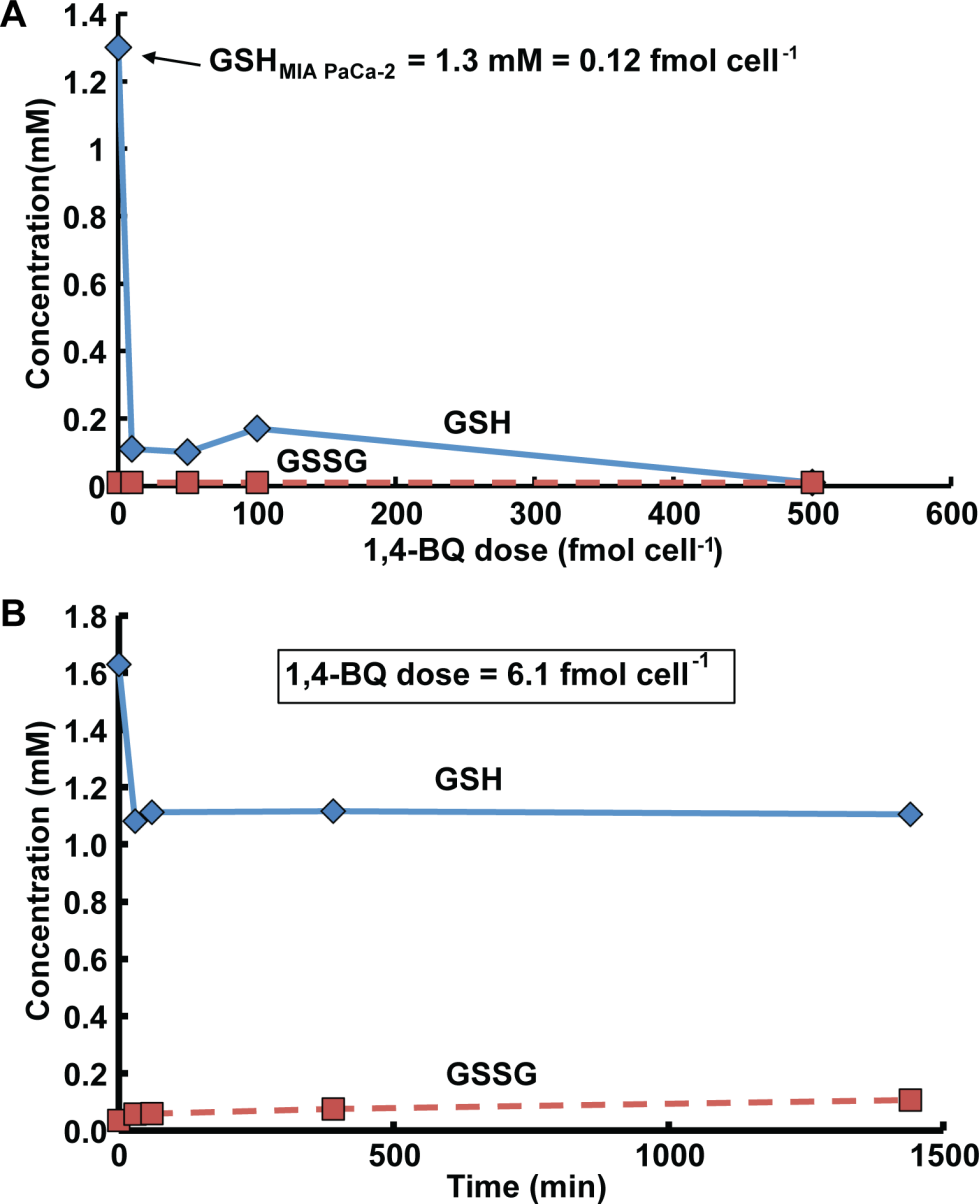
Glutathione is not depleted with 1:1 stoichiometry upon exposure of MIA PaCa-2 cells to 1,4-BQ. **(A)** Intracellular concentration of GSH and GSSG in MIA PaCa-2 cells after a 30-min exposure to different doses of 1,4-BQ. The basal level of GSH in MIA PaCa-2 cell is 0.12 fmol cell^-1^ or 1.3 mM, assuming a uniform intracellular distribution and an intracellular volume of 2.03 pL cell^-1^, Table 1. To deplete 90% of the basal intracellular GSH a large excess of 1,4-BQ (10 fmol cell^-1^) is required. Additional depletion up to 99% requires ≈400 fold excess of 1,4-BQ. This implies that 1,4-BQ most likely reacts with extracellular targets; the fraction that enters the cells reacts with the multitude of targets available in the intracellular space. GSSG is not significantly produced (statistically) in these experiments. **(B)** Intracellular concentration of GSH and GSSG up to 24 h after exposure to a bolus of 1,4-BQ. Following exposure of MIA PaCa-2 cells to 1,4-BQ (6.1 fmol cell^-1^), an immediate 31% depletion in GSH levels is observed. There is no recovery of GSH for at least 24 h after exposure. There is little generation of GSSG, indicating negligible generation of H_2_O_2_. On a mol cell^-1^ basis, only 1 out of 100 of the molecules of 1,4-BQ that were present at the start of the exposure reacted with GSH; This demonstrates that GSH is not the exclusive target for 1,4-BQ. Shown here are typical experiment results, *n* = 3. The coefficient of variation in the GSH and GSSG measurements is 13%.

For exposure-science to move ahead in this 21^st^ century, it is imperative to create quantifiable causal relationships between agent and biological target. Here, the use of mol cell^-1^ as a dosing metric to aid in this process was investigated. 1,4-BQ is known to react with many nucleophilic moieties in proteins and small molecules, intracellularly as well as extracellularly. However, the main target(s) for toxicity has(have) yet to be identified. Glutathione was investigated as a potential target of 1,4-BQ, which can be directly related to the observed toxicity. A dose-dependent depletion of intracellular GSH in MIA PaCa-2 cells upon exposure was observed, Fig 6A. As expected, only a small accumulation of GSSG was observed over 24 h, Fig 6A. There was no detectable effect on the intracellular concentration of GSH or GSSG due to exposure to DMSO (vehicle) alone, S2 Fig.

The dose (mol cell^-1^) of 1,4-BQ required to achieve ~90% depletion of GSH was about 100 times the total amount of GSH in a cell. There is no 1:1 relationship between the amount of added 1,4-BQ and GSH depletion; this indicates the prevalence of multiple reactions of 1,4-BQ with targets other than GSH, Fig 6B. The kinetics of the depletion of intracellular GSH was rapid and no recovery was observed in the 24 h following exposure, Fig 6B. Even though GSH is rapidly depleted, a significant decline in clonogenic survival is only observed when intracellular GSH values are <1% of initial values, S3 Fig. More importantly, the dose of 1,4-BQ per cell required to observe significant toxicity is many orders of magnitude greater than the amount of intracellular GSH. A consideration not addressed here is the amount of 1,4-BQ “lost” to other compartments, e.g. to the cell culture medium, cell culture vessel, evaporative losses, and other avenues, i.e. the 1,4-BQ that is not associated with cells. These potential routes for “loss” of xenobiotics in cell culture is the central theme of [5]. The reactivity/association of 1,4-BQ with the non-cellular components of experiments would greatly diminish the amount actually associated with cells, consistent with our observation. However, mol cell^-1^ provides considerably more information than nominal concentration and is a better starting point to investigate and account for the “lost” 1,4-BQ.

### Intracellular volume affects the apparent toxicity of 1,4-BQ

**Fig 7:**
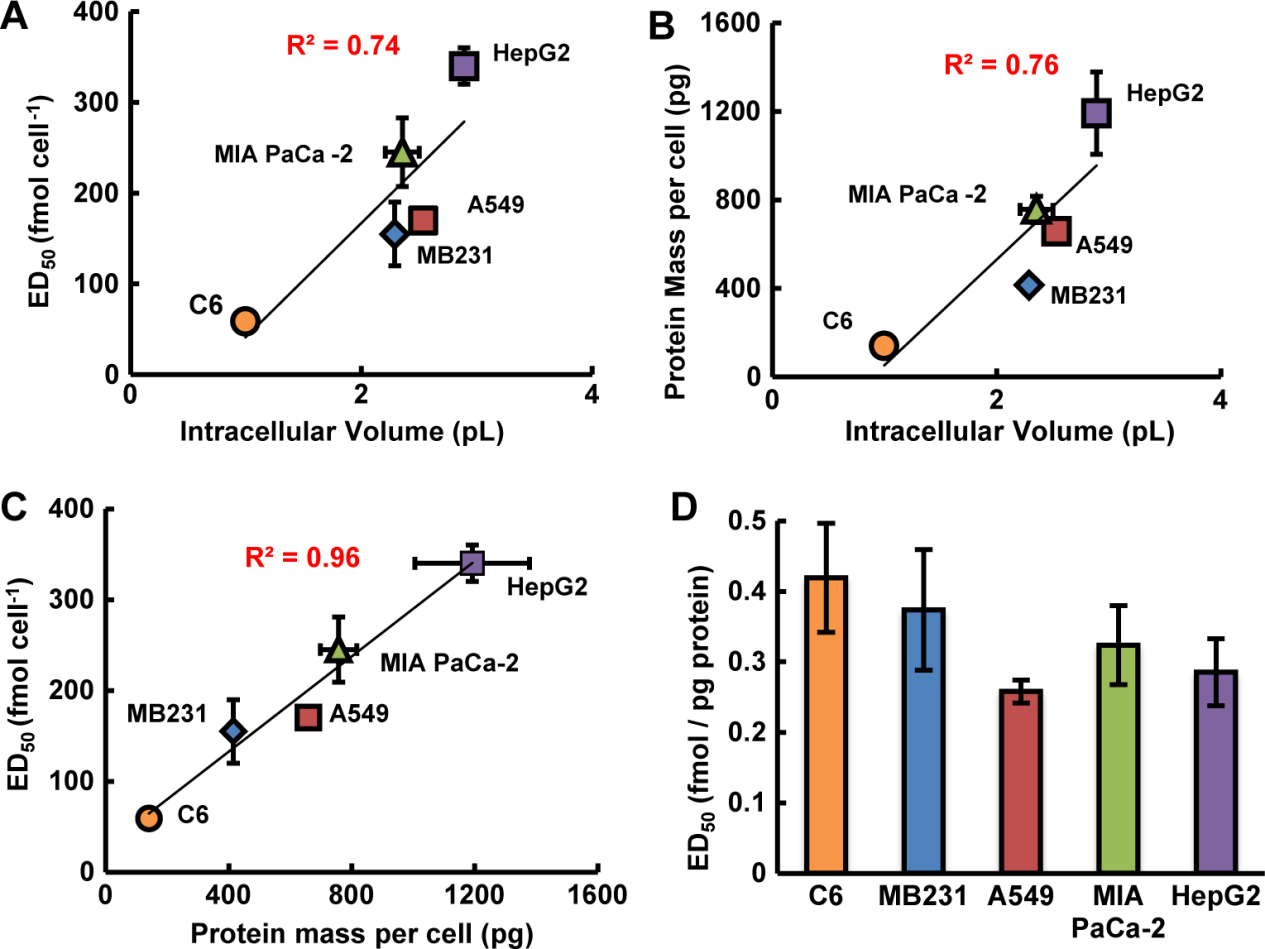
ED_50_ of 1,4-BQ correlates directly with intracellular volume and mass of protein per cell for C6, MB231, A549, MIA PaCa-2, and HepG2 cell lines. **(A)** The dose of 1,4-BQ (mol cell^-1^) at which 50% clonogenic survival was observed for each cell type is plotted vs. the measured intracellular volume (Table 1). The correlation coefficient R^2^ is 0.74. Each cell line has *n* = 2 for biological replicates, *n* = 3 within each replicate. The measured intracellular volume represented is the mean of two different methods of measuring intracellular volume. Each cell line was measured (*n* = 3) using a Z2 Coulter Counter and a Moxi Z Mini Automated Cell Counter in ISOTON II Diluent (Beckman Coulter, Inc.). Error bars represent the standard error of the mean; some error bars are smaller than the symbol. **(B)** Mass of protein per cell directly correlates with intracellular volume (R^2^ = 0.76). Protein content was measured in the five cell lines used (C6, MDA-MB231, A549, MIA PaCa-2, and HepG2) by the SDS-Lowry protein assay. Some of the uncertainties in protein mass per cell are smaller than the symbols. Error bars represent the standard error of the mean. Each protein measurement has *n* = 3 for biological replicates, *n* = 3 within each replicate. **(C)** The ED_50_ of 1,4-BQ for C6, MB231, A549, MIA PaCa-2, and HepG2 cells (mol cell^-1^) is plotted vs. measured protein mass (pg) per cell. The correlation coefficient R^2^ is 0.96. Error bars represent the standard error of the mean. **(D)** The ED_50_ of 1,4-benzoquinone is expressed as fmol of 1,4-benzoquinone per pg protein for a cell. Each protein measurement has *n* = 3 for biological replicates, *n* = 3 within each replicate. Error bars represent the propagation of error as determined from the standard error of the means for both protein measurements and ED_50_ of 1,4-BQ. When dose of 1,4-BQ is expressed as fmol pg^-1^, there was no statistical difference in the ED_50_ of 1,4-BQ observed across the different cell lines. ANOVA showed *p* > 0.05 for all comparisons.

**Fig 8:**
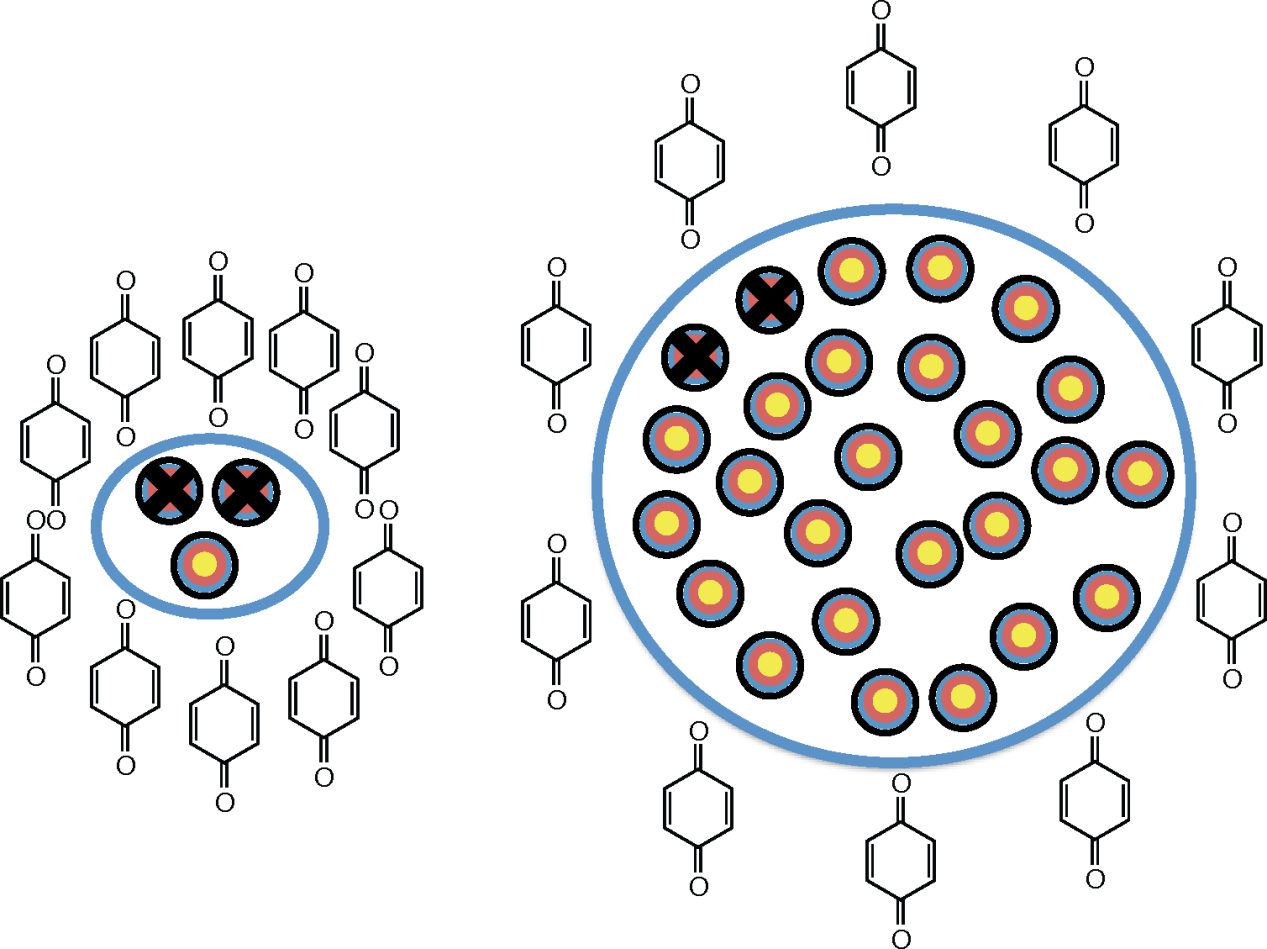
Depiction of target theory and exposure to 1,4-BQ. The ten quinone moieties shown in each scenario represents the same mol cell^-1^. Here we assume that there are a certain number of sensitive/reactive “targets” within cells and the number of targets is proportional to intracellular volume. Damage to some fraction of those targets will produce a biological effect. Because larger cells have a greater number of targets, more 1,4-BQ will be required to produce the same biological effect as observed with smaller cells.

Because the presumed principal mode of toxicity for 1,4-BQ is to form stable covalent bonds with cellular components, it is hypothesized from target theory that larger cells with more intracellular components may be more resistant to exposures of 1,4-BQ than smaller cells. To test this hypothesis and the usefulness of mol cell^-1^ in establishing this relationship, the toxicity of 1,4-BQ to five different cell lines with different intracellular volumes (Table 1) was determined, Fig 7 panels A-D. A strong linear correlation between ED_50_ (clonogenic assay) of 1,4-BQ with measured intracellular volume was observed (R^2^ = 0.74), Fig 7A. As expected, the mass of protein per cell directly correlates with the intracellular volume (R^2^ = 0.76) [16], Fig 7B. A very strong linear correlation between ED_50_ of 1,4-BQ with the measured mass of protein per cell of the different cell lines was observed (R^2^ = 0.96), Fig 7C. This is consistent with there being more targets (*i*.*e*. proteins) in larger cells than smaller cells that must be modified by 1,4-BQ to produce reproductive cell death, Fig 8.

A standard approach for normalization of a biochemical or biological consequence, here ED_50_, is to normalize the results to protein mass as determined with a protein assay. In the experiments of Fig 7 when ED_50_ is normalized to mass of protein (here pg of protein), all cells appear to be equally susceptible to 1,4-BQ, Fig 7D. Normalization to mass of protein would be considered a biochemical normalization, whereas normalization to per cell would be a biophysical normalization. As demonstrated here, these two different normalizations can provide very different but complementary information.

## Discussion

Cell culture is a widely used research tool in the life sciences. It is used to study the basic biology of health and disease and is essential in characterizing the biochemical and biological effects of a wide range of xenobiotics, including studies on the efficacy and toxicity of new therapeutics for the treatment of disease. Here, we propose a simple, straightforward, easy to implement, and cost-effective strategy to reconsider how dose and exposure are used and reported in typical cell culture experiments. This approach can greatly expand the quantity and quality of the information obtained in many settings. In this work, we provide examples of how mol cell^-1^ is a dosing metric that is less affected by physical parameters, such as volume of medium and number of cells, because it incorporates more information about the physical set-up within the metric. In doing so, it yields more consistent dose-response information that is not easily obtained by using other dosing metrics. This information can help to understand both biological (e.g., clonogenic survival) and biochemical (e.g., ATP content) endpoints. We anticipate that this approach may also be useful for experiments with non-mammalian models of toxicity e.g. yeast, bacteria, and perhaps even *C*. *elegans*, zebra fish, and drosophila. However, if the ratio of extracellular (or extra-organism) volume containing the xenobiotic to intracellular (or intra-organism) volume is extremely large, then mol cell^-1^ or mol organism^-1^ will be inappropriate as the amount of substance per cell/organism could be far too great for the organism to make any significant change in the steady-state level of available xenobiotic, or availability is diffusion-limited. Then different aspects of exposure would need to be considered and be more appropriate.

The concept of mol cell^-1^ extends not only to toxicant or drug, but also to agents used as biochemical tools to determine the fundamental mechanisms of biochemical and biological pathways. Oligomycin A is one such agent used to study mitochondrial function. As an example, oligomycin A is part of the mitochondrial stress test kit that Seahorse Bioscience provides for use with their XF analyzers to profile mitochondrial function. Here we show that the same initial concentration of oligomycin A has a different effect on the intracellular ATP concentration when varying numbers of cells are present. However, when applied dose is specified as mol cell^-1^ this caveat does not apply.

### Different experimental platforms can result in a wide range of exposures

**Fig 9:**
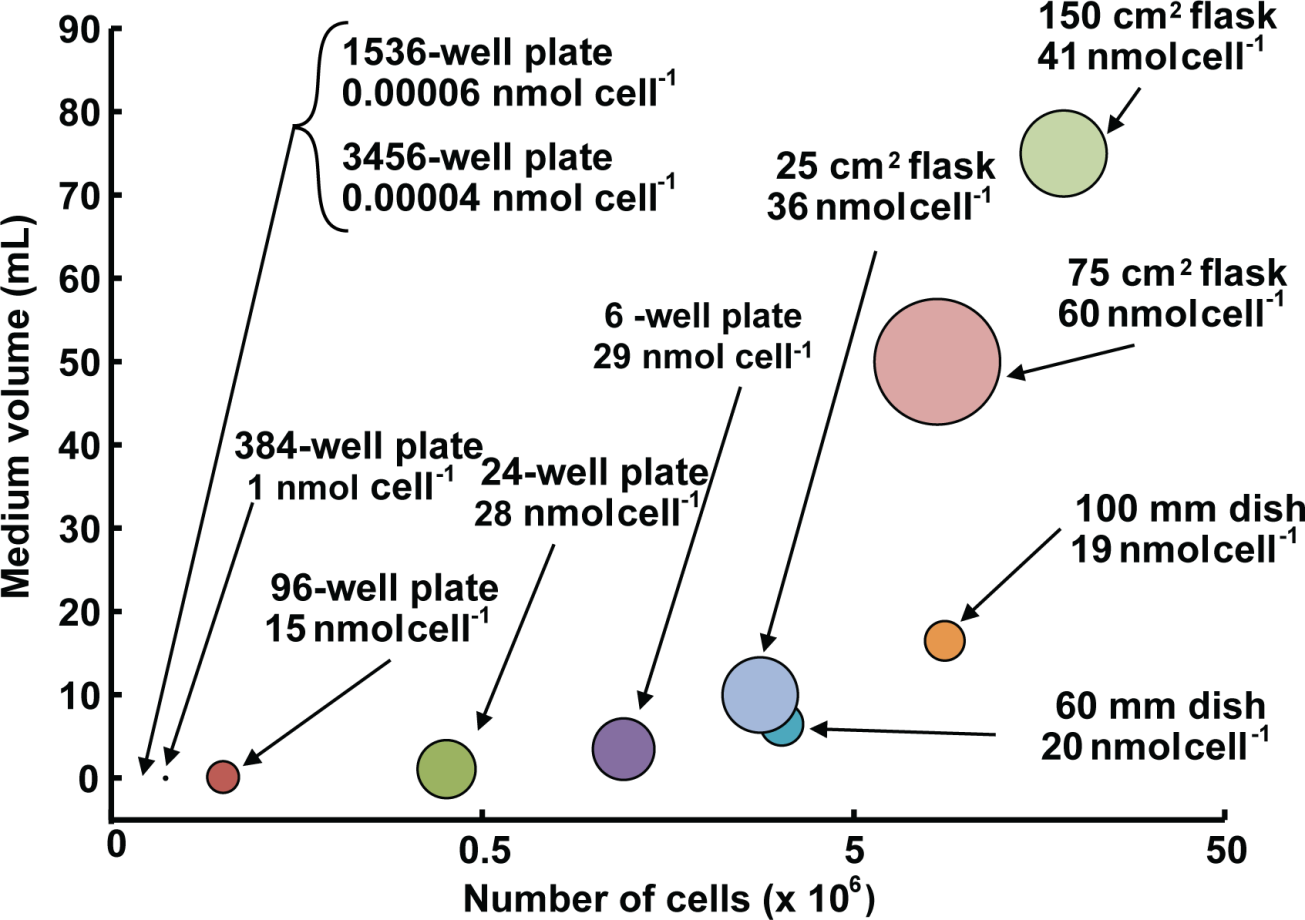
Exposure to xenobiotics when specified as mol cell^-1^ varies greatly when using different experimental platforms for cell culture. Hypothetical cell culture experiments are shown where general recommendations, such as from Invitrogen (Life Technologies, Grand Island, NY), are followed for: seeding density, estimated number of cells at confluence, and media volume when using different cell culture vessels. In all hypothetical experiments, cells were exposed to an initial concentration of 10 μM xenobiotic in the medium. However, this leads to a wide range of doses when converted to mol cell^-1^. The size of each data point (circle) is proportional to the dose in units of nmol cell^-1^; the larger the area of the circle, the higher the exposure. Note that for platforms commonly used in wet bioscience laboratories, dose per cell varies over a range of 60-fold; if platforms used for high-throughput-screening are included (1536- and 3456-well plates) then dose per cell varies over a 10^6^-fold range, despite the initial molar concentration of xenobiotic being the same for each experimental platform.

We demonstrate that mol cell^-1^ can reconcile what appear to be very different results from what would seem to be the same experiment, but performed under different physical configurations. Nowhere is this more important than in the state-of-the-art high-throughput screening of new drugs and toxicants. There is a wide range of experimental platforms (traditional culture vessels with robotic operation to low volume, low cell number multi well plates) available for these types of studies. If not controlled for, these distinct platforms can provide different experimental results under seemingly similar conditions, Fig 9. Note that for platforms commonly used in wet bioscience laboratories, dose per cell varies over a range of 60-fold under typical exposure conditions and an identical initial concentration. If platforms used for high-throughput-screening are included, then dose per cell varies over a 10^6^-fold range. When designing experiments using different platforms, exposure as mol cell^-1^ will reduce costs (S1 Impact), improve reproducibility, and allow direct comparison between experiments.

### The case of H_2_O_2_ and mol cell ^-1^ or mol cell ^-1^ s ^-1^ as a metric for exposure

The concept of toxicity as a function of cell density has been noted before; an example is hydrogen peroxide (H_2_O_2_) [28, 29, 30], and references therein. Hydrogen peroxide can bring about both reversible and irreversible changes in covalent bonds. As a cellular toxin, the mechanism of toxicity for H_2_O_2_ involves oxidation of proteins, lipids, DNA, as well as the intracellular redox buffer [31, 32, 33]. In different settings these mechanisms have different degrees of importance towards the overall toxicity of H_2_O_2_. Recently Gülden et al. described the toxicity of bolus additions of H_2_O_2_ to cells in culture on the basis of per cell exposure, i.e. μmol H_2_O_2_ per 10^7^ cells [29]. Their work was derived from earlier work by Spitz et al. [28] who concluded that the primary descriptor of toxicity of H_2_O_2_ is nmol H_2_O_2_/mg cell protein. As expected, when different physical setups of experiments were investigated they observed that the initial extracellular concentration of H_2_O_2_ (molarity) did not necessarily correlate with the observed toxicity. However, their data could be easily interpreted when dose was specified as nmol H_2_O_2_ /mg cell protein; for a given cell line, this is proportional to mol cell^-1^, Fig 7B and Table 1.

This concept applies not only to direct additions of H_2_O_2_ to cells in culture but also to sources that generate a flux of H_2_O_2_, e.g. the glucose/glucose oxidase system [34,35] or the oxidation of ascorbate in cell culture [36]. A central consideration in designing successful experiments is knowledge on the flux (mol cell^-1^ s^-1^) of H_2_O_2_ in a set of experiments. In all such experiments it is important to determine (or verify) the flux of H_2_O_2_ from sources of H_2_O_2_ on a mol cell^-1^ s^-1^ basis. It is only with this approach that repeatability can be achieved and the best understanding of experimental data can be obtained. With this approach the transition from observational biology to quantitative biology is possible [16, 37, 38].

The realization by a very few researchers over 20 years ago that mol cell^-1^ (nmol H_2_O_2_ /mg cell protein) of H_2_O_2_ is the coin-of-the-realm for specifying dose for exposure to H_2_O_2_ remains largely unappreciated. Literally hundreds of scientific papers each year employ bolus addition of H_2_O_2_ to the medium of cells in culture reporting only the initial concentration of H_2_O_2_ in the medium. The vast majority of reports use initial molarity to specify dose, often without information on number of cells or volume of medium, i.e. cell density (cells L^-1^) limiting the information available in the data. Different physical setups of experiments will result in vastly different lifetimes of H_2_O_2_ [38] and most probably different results.

We demonstrate here that expression of dose as mol cell^-1^ has application beyond exposures to H_2_O_2_. Dose of a xenobiotic as mol cell^-1^ can be easily implemented by making note of physical parameters used in the experiment, such as volume of medium and number of cells. Utilizing this information in a dosing metric when setting up experiments allows for increased consistency in the larger body of information available in experimental data.

### Target theory

Target theory is a concept highly applicable when interpreting results from experiments using mol cell^-1^ as the dosing metric. As first described by Lea in 1946 [39], target theory assumes there to be a certain number of sensitive points or “targets” within cells. Damage to a certain percentage of those targets will cause the cell to undergo a response. The experiments with bolus *vs*. sequential additions of 1,4-BQ provide an excellent example of “hits” to a target accumulating to a critical percentage leading to biological effects, Figs 5 and 8. Here we assume that the “damage” to critical targets is not reversible. Our experimental results with the various cell lines (varying cell volumes and thus a varying number of targets for 1,4-BQ, Figs 7 and 8) is consistent with different cell lines having a distinct number of targets. A certain fraction of these targets need to be hit to initiate an effect. This results in a different threshold for the amount of 1,4-BQ required amongst the cell lines before a specific toxicological endpoint is observed. The observation of a strong linear correlation of toxicity, ED_50_, *vs*. cell size or protein mass cell^-1^ is consistent with the principles of target theory, assuming the number of targets susceptible to change by 1,4-BQ is proportional to cell volume or protein mass cell^-1^, Fig 7. This is in a way parallel to how administration of many drugs or exposure to toxicants is considered, i.e. the mass of the patient or area, e.g. per m^2^.

The cell lines tested have quite different tissue origins (Table 1) and characteristics that contribute to the consequences that ensue upon exposure to a xenobiotic. However, the results of Fig 7 indicate that elements of target theory can be used to understand some aspects of these effects. This concept also underpins mol cell^-1^ as a unique approach to compare the availability of targets for a xenobiotic in various cell lines.

### Bolus vs. sequential additions

Because of the rapid reaction of 1,4-BQ with thiols and amines, a bolus addition could have different apparent cellular toxicities, depending on the particular assay employed. We found that giving a single bolus dose of 1,4-BQ or 12 sequential smaller doses, which yielded the same total dose, produced the same toxicity, as seen by clonogenic survival, Fig 5. Parallel observations using activation of signaling pathways have been made with reactive lipid-derived electrophiles attesting the generality of this mechanism [40].

However, changes in membrane-integrity upon exposure to 1,4-BQ, as seen by the trypan blue assay, showed significant differences in apparent toxicity between the two methods of exposure. These observations indicate to the importance of time in addition to dose in understanding mechanisms of toxicity. The time frame for the 12 sequential additions, 4 h, was much less than the doubling time for MIA PaCa-2 cells, ≈24 h. From this experiment the short time and lower amount of 1,4-BQ allowed time for “repair” of the membrane damage as seen by trypan blue exclusion. However, the damage as seen by the clonogenic assay was apparently cumulative. One can speculate that if the time frame for the sequential addition was on the order of, or greater than, the cell doubling time, then less toxicity would have been observed.

The choice of assay to assess toxicity depends on the goals of the experiment and the information sought. Here we see that clonogenic survival is dependent on total dose, but membrane integrity is not associated with total dose under these experimental conditions. These apparent differences in toxicity are revealed by using mol cell^-1^ as the dosing metric providing the best information to understand the mechanism of lethality.

### Limitations

In this work mol cell^-1^ is the nominal dose of a xenobiotic applied in the medium normalized to the number of cells present at time of exposure. This is not intended to imply that it is the dose associated with the cell, i.e. the cell burden or internal concentration. Mol cell^-1^ may not provide an advantage in all experimental settings. The biochemical and biological effects of xenobiotics that bind reversibly to cellular targets, such as hydrogen bonding, are governed by the equilibrium constant for the binding and, of course, mass action. For xenobiotics that act *via* “equilibrium” reactions, especially those with relatively large dissociation constants, the traditional dosing metric of initial concentration in the medium may be a useful dose metric. However for tight complexes, i.e. small dissociation constants, mol cell^-1^ may be a very informative metric.

Here we propose that mol cell^-1^ can be especially valuable with agents that bring about “irreversible” changes in cells. As examples to test our hypothesis, we examined the classic electrophile 1,4-BQ, which makes irreversible covalent bonds with cellular components, and oligomycin A, which forms a tight complex with mitochondrial H^+^-ATP synthase [24, 26, 27]. 1,4-Benzoquinone as an electrophilic species reacts readily with thiol- and amine-containing species forming covalent bonds, S1 and S3 Figs [41, 42, 43]; biological effects can ensue [44, 45, 46]. Mol per cell is a better approach than nominal concentration (i.e. initial concentration) to specify dose for these two quite different xenobiotics. We foresee that this approach to specify dose will be valuable all across cell culture for a wide range xenobiotics, be they toxicants, drugs, or standard biochemical tools. However, neither nominal concentration nor mol cell^-1^ addresses the amount lost due to chemical reactions with or binding to medium components or even the cell culture vessel itself as well as other possible routes, as addressed thoroughly by Groothuis et al. [5].

The only additional laboratory effort to arrive at mol cell^-1^ is to count the number of cells in an experiment. This requires little additional effort and resources. However, if the cell number changes significantly during an experiment, then additional considerations will be needed as the amount of xenobiotic per cell may not be easily estimated.

### Recommendations

The vast majority of biochemical assays require some sort of normalization. For example enzyme assays are most often reported in some appropriate units that are normalized to amount of protein; examples are the assays for glutathione peroxidase activity [47], catalase activity [48], and superoxide dismutase activity [49]. These types of results from cell culture experiments can also be normalized to the number of cells [16, 38]. Normalizing to protein content is considered to be a biochemical normalization, whereas normalizing to number of cells is a biophysical normalization. These two approaches provide different, but complementary information. Nominal extracellular concentration is not a normalized parameter. However, specifying dose or exposure in mol cell^-1^ (i.e. (nominal moles (or mass) of xenobiotic)/(number of cells)) offers a scalable parameter that can be used to design experiments and help interpret a wide variety of experimental results. Clearly this parameter will be an upper limit to the number of moles of xenobiotic, or downstream products, associated with a cell; some fraction will not be in/on the cells, but rather may be associated with media or experimental apparatus or lost through other mechanisms [5]. We recommend that when reporting on experiments that employ cell culture, researchers should specify the dose of xenobiotics both in traditional initial concentration units (nominal concentration) as well as mol cell^-1^ or mass cell^-1^ (a normalized nominal amount) to provide maximum information. Employing this dosing metric can be accomplished with little additional effort or resources. We also recommend cell number be determined prior to treatment in a representative flask (or well in a plate), as opposed to after treatment. This insures that all cells that are present at the time of exposure are accounted for and includes those cells that may undergo cell death during exposure, which would not be taken into account if cells are counted following the exposure. The method and time point of counting cells should always be clearly reported to reduce ambiguity and allow for replication of experiments.

## Conclusions

In this study, some of the advantages and limitations of mol cell^-1^ *vs*. initial molarity of the agent in the medium were examined. We conclude:

1. Dose expressed as mol cell^-1^ allows direct comparisons between different experimental conditions and can more accurately report the actual exposure compared to nominal (initial) concentration in the medium, Figs 1–4.
2. Dose/exposure as mol cell^-1^ can provide insightful information when exposures in cell culture are not from a single bolus addition, Fig 5.
3. The biological effects of xenobiotics that make irreversible covalent bonds depend on cell size and protein content, which is in line with concepts of target theory, Figs 6–8.
4. Mol cell^-1^ provides a scalable metric of dose that allows for successful transition between different cell culture platforms; *i*.*e*. all the information needed is included in this metric to scale experiments to different platforms that require differing number of cells and volumes of medium, Fig 9.

Data presented here make a compelling case to include mol cell^-1^ along with traditional nominal concentration units when specifying dose in cell culture studies.

## Supporting Information

S1 Fig Products formed upon the Michael addition reaction of thiols and primary amines to 1,4-benzoquinone. (PDF)

S2 Fig Exposure to DMSO, the vehicle for 1,4-BQ, does not affect the intracellular concentration of GSH or GSSG. (PDF)

S3 Fig The intracellular concentration of GSH correlates poorly with clonogenic cell survival upon exposure to 1,4-benzoquinone. (PDF)

S1 Impact Impact. (PDF)
