# Supplementary material for: Moles of a Substance per Cell Is a Highly Informative Dosing Metric in Cell Culture
Source: PLoS One. 2015 Jul 14;10(7):e0132572. doi: 10.1371/journal.pone.0132572 (PMC4501792; doi:10.1371/journal.pone.0132572)
Supplement: S1 Fig — (PDF) [file pone.0132572.s001.pdf]

S1_Figure

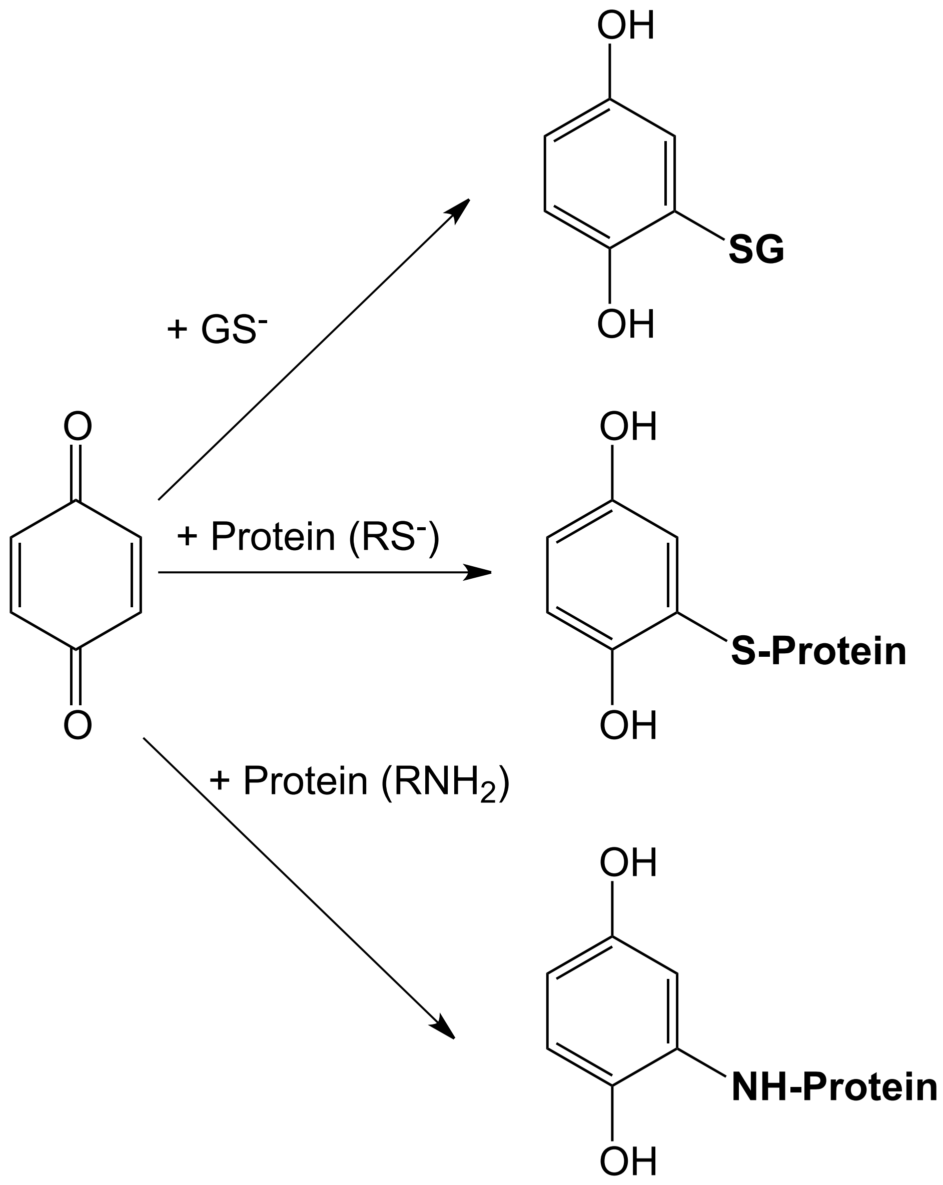

Supporting Information, Figure S1. Products formed upon the Michael addition reaction of thiols and primary amines to 1,4-benzoquinone. The reductive addition reaction of thiols, to 1,4-BQ is rapid, with an observed second-order rate constant on the order of 10^6 M^{-1} s^{-1} at near-neutral pH [39, 40]. The reaction is pH-dependent with the ionized thiol or non-protonated amine being the effective nucleophiles. The rate constant for the reductive addition of amines is considerably smaller, on the order of 10^{-2} M^{-1} s^{-1} in pH 7.4 buffer [40].
