# Supplementary material for: Moles of a Substance per Cell Is a Highly Informative Dosing Metric in Cell Culture
Source: PLoS One. 2015 Jul 14;10(7):e0132572. doi: 10.1371/journal.pone.0132572 (PMC4501792; doi:10.1371/journal.pone.0132572)
Supplement: S2 Fig — (PDF) [file pone.0132572.s002.pdf]

**S2_Figure**

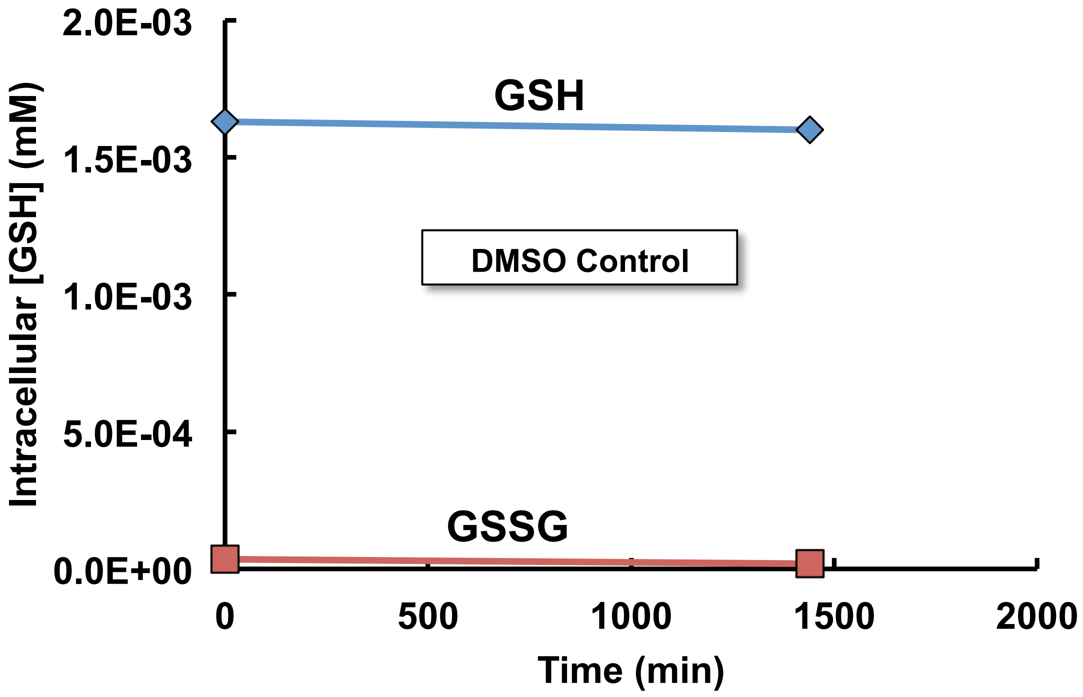


**Supporting Information Figure S2. Exposure to DMSO, the vehicle for 1,4-BQ, does not affect the intracellular concentration of GSH or GSSG.** The protocol to expose cells to DMSO alone were identical to those used to expose cells to 1,4-BQ using DMSO as the vehicle.
