# Supplementary material for: Moles of a Substance per Cell Is a Highly Informative Dosing Metric in Cell Culture
Source: PLoS One. 2015 Jul 14;10(7):e0132572. doi: 10.1371/journal.pone.0132572 (PMC4501792; doi:10.1371/journal.pone.0132572)
Supplement: S3 Fig — (PDF) [file pone.0132572.s003.pdf]

S3_Figure

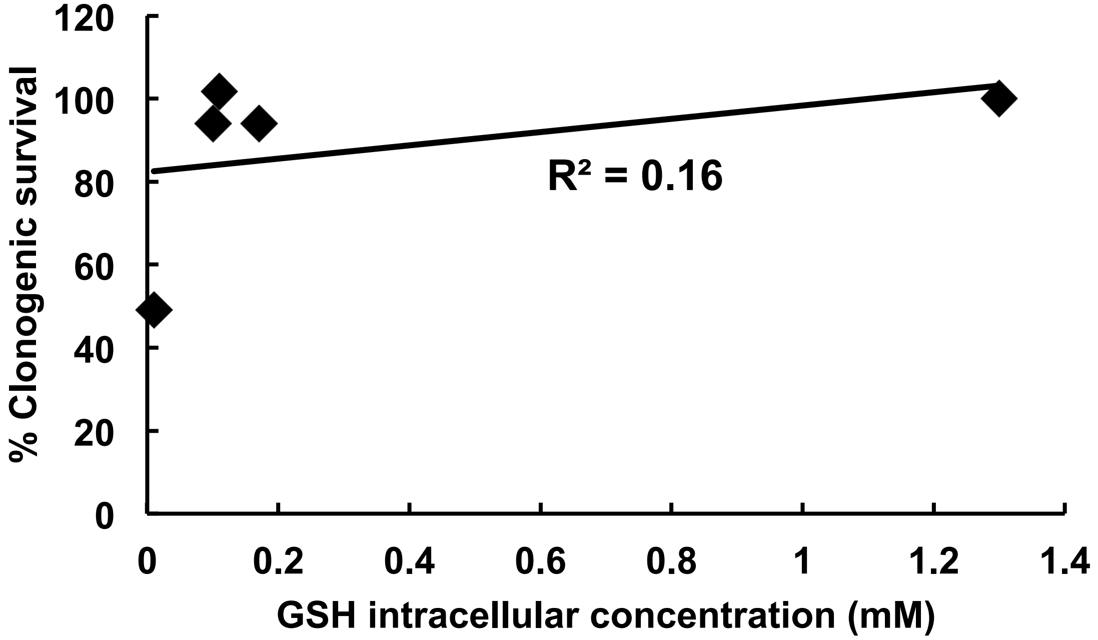

Supporting Information Figure S3. The intracellular concentration of GSH correlates poorly with clonogenic cell survival upon exposure to 1,4-benzoquinone. Immediately after a 30 min exposure to 1,4-BQ a portion of the cells were plated for clonogenic cell survival. The remaining cells were utilized for the measurement of GSH and GSSG with HPLC-BDD. Experiments were performed with MIA PaCa-2 cells.
