# Supplementary material for: Moles of a Substance per Cell Is a Highly Informative Dosing Metric in Cell Culture
Source: PLoS One. 2015 Jul 14;10(7):e0132572. doi: 10.1371/journal.pone.0132572 (PMC4501792; doi:10.1371/journal.pone.0132572)
Supplement: S1 Impact — (PDF) [file pone.0132572.s004.pdf]

S4_Impact

A. Information expansion

The major impact of the use of mol cell^{-1} to specify dose along with traditional methods is that significantly more information will be available in the data from many cell culture experiments. Estimating impact on the potential information content is somewhat subjective, but if we very conservatively assume that in a year that researchers gain as little as 5 – 10% more information each year, then after a decade the increased amount of information available from these experiments would be in the range of:

$$\textit{Increased Information} = (1.05)^{10} \text{ to } (1.10)^{10} = 1.6 \text{ to } 2.6$$

that is, the amount of new information would be approximately doubled.

If one takes that view that more information is gained from each set of successful experiments that employ cell culture, and very conservatively that approximately 12 sets of experiments are completed each year by a researcher and in each set of experiments there is 1 – 5% more information in the data, then the increased amount of information gathered over a decade would be:

$$\textit{Increased Information} = (1.01)^{(10 \times 12)} \text{ to } (1.05)^{(10 \times 12)} = 3 \text{ to } 350$$

Whether considered from the view of increased information gained over a year, or per experiment, the return on investment over a decade could be rather substantial.

B. Resource conservation.

The time and resources saved because of failed experiments due to too few cells, too many cells, too little xenobiotic or too much xenobiotic and all the possible combinations needed to tease out the information sought in a set of experiments can also be substantial. If for simplicity we assume a flat budget over a decade for cell culture experiments in a laboratory and the savings in time and resources is 10 – 20% each year, then over a decade the total savings is simply 10 - 20%, which is comparable to getting 1 -2 years of “free experiments”.

However, if the success rate increases by 10 - 20%, then rather than 12 successful experiments each year, this could increase to 14, then the information available after a decade would be further increased, for example

$$\textit{Increased Information} = (1.01)^{(10 \times 14)} \text{ to } (1.05)^{(10 \times 14)} = 4 \text{ to } 926$$

In our experience the above are conservative estimates of the advantages. Both the savings in resources as well as the increased information available make a compelling case to include mol cell^{-1} along with traditional concentration units when specifying dose in cell culture studies.

Doskey CM, van ‘t Erve TJ, Wagner BA, Buettner GR. *Moles of a Substance per Cell is a Highly Informative Dosing Metric in Cell Culture*
